# Surgery at the Base

**Published:** 1919

**Authors:** 


### SURGERY AT THE BASE

Compilation of Responses by Base Hospital Staffs to Questionnaire sent out by Research Committee. Questionnaire prepared by Brigadier-General J. M. T. Finney and Colonel G. W. Crile. Compilation of responses made by Major T. W. Burnett, M. C.

### RESPONSES TO QUESTIONNAIRE ON "SURGERY AT THE BASE"

I. General.

Q. — 1. **What is the total number of admissions to your hospital?**

2. **What is the number of deaths? How many deaths from pneumonia?**

3. **What is the number of surgical cases? Number of deaths? Among these how many were caused by pneumonia?**

| HOSPITAL | Total Admissions | Total Deaths. | Total Deaths Pneumonia | Surgical Cases. | Surgical Deaths. | Surgical Pneumonia Deaths. |
|---|---|---|---|---|---|---|
| A.R.C.Mil.2 | 8,150 | 151 | 24 | 5,457 | 76 | 16 |
| Base 49. . . | 4,303 | 83 | 39 | 2,265 | 34 | 5 |
| " 114. . . | 12,808 | 90 | 36 | 9,000 | 50 | 8 |
| " 43. . . | 5,703 | 97 | 38 | 3,114 | 39 | 5 |
| " 115. . . | 6,140 | 41 | 4 | 3,958 | 27 | 8 |
| " 30. . . | 6,994 | 162 | 129 | 2,927 | | 4 |
| " 8. . . | 31,500 | 186 | 79 | 19,579 | 46 | 9 |
| " 11. . . | 5,668 | 48 | 21 | 3,311 | 27 | 5 |
| " 48. . . | 4,707 | 83 | 28 | 2,890 | 54 | 1 |
| " 61. . . | 2,693 | 70 | 19 | 1,526 | 35 | 5 |
| Center 50. . | 7,461 | 267 | 165 | | | |
| Base 22. . . | 16,418 | 140 | 90 | 6,101 | 24 | 8 |
| " 35. . . | 6,155 | 68 | 35 | 1,771 | 32 | 5 |
| " 47. . . | 4,400 | 114 | 72 | 2,312 | 24 | 8 |
| " 12. . . | 55,601 | 404 | 68 | 28,758 | 295 | 0 |
| " 62. . . | 5,349 | 30 | 21 | 426 | 4 | 2 |
| " 38. . . | 5,743 | 55 | 20 | 2,625 | 32 | 3 |
| A.R.C.Mil.1 | 24,543 | 467 | 37 | 22,954 | 446 | 23 |
| Base 5. . . | 21,668 | 301 | 95 | | | |
| " 3. . . | 8,648 | 168 | 105 | 3,379 | 45 | 5 |
| " 2. . | 21,836 | 237 | 62 | 8,409* | 164 | 7 |
| " 4. . . | 68,513 | 710 | | | | |
| " 10. . . | 45,756 | 533 | 94 | 23,421 | 247 | |
| " 14. . . | 5,099 | 90 | 44 | 2,045 | 40 | 13 |
| " 19. . . | 10,287 | 69 | 33 | | | |
| N° no. . . | 5,460 | 88 | 17 | 2,558 | 43 | 5 |
| Base 6. . . | 23,288 | 420 | 284 | 17,466 | 77 | |
| Evac. 1 . . | 7,104 | 364 | 2 | 7,050 | 359 | 0 |
| Base 60. . . | 3,509 | 19 | 14 | 1,593 | 3 | 0 |
| " 31. . . | 8,645 | 120 | 46 | 4,869 | 57 | 11 |
| " 15. . . | 30,000 | 428 | 176** | | | |
| " 20. . . | 7,500 | 61 | 12 | 2,408 | 49 | 7 |
| " 41. . . | 4,652 | 63 | 21 | 3,458 | 42 | 14 |
| " 7. . . | 5,504 | 49 | 33 | 2,223 | 20 | 6 |
| " 34. . . | 8,396 | 108 | 43 | | | |
| Totals . . | 500,201 | 6,378 | 2,007 | 199,943 | 2,391 | 183 |

Death Rate to Total Admissions . . . . . . based on 35 hospitals. 1.27 %
" " (pneumonia) to Total Admissions. " " 34 " .40 %
" " in Surgical Cases. . . . . . . . " " 28 " 1.22 %
" " (pneumonia) in Surgical Cases . " " 26 " .09 %

Note : — (Several incomplete returns not included in perce ıtages).
* " Including Gas Cases ".
** " Confirmed by Autopsy ".

Q. — 4. **How long should abdominal cases be held at the front before transportation?**

A. — Concensus : Until danger from acute peritonitis is past — from ten days to two weeks, depending upon nature of wound and operation procedure.

Q. — 5. **How do through-and-through chests travel?**

A. — No. of Votes : Badly, 8. Well, 23.

Expression of remainder :

1. Well after one week.
2. Shrapnel poorly — perforating rifle bullet wounds well.
3. Non-operated cases without shock, well.
4. Operated cases before healing or those with severe intra-thoracic conditions, badly.

Remark : Difference of opinion evidently accounted for by results due to factors of transportation and sector conditions.

Q. — 6. **What type of cases are most injured by travel?**

A. — *a*) Penetrating abdominal.
*b*) Muscle wounds in locations favoring developing of gas infection — buttocks, etc.
*c*) Compound comminuted fractures of femur, with or without knee-joint involvement.
*d*) Severely shocked cases.
*e*) Brain cases.
*f*) Sucking chest wounds.
*g*) Complicating pneumonias.
*h*) Fresh amputations.
*i*) All improperly splinted fractures.
*j*) High-explosive chest injuries.
*k*) Operated abdominal wounds.
*l*) All serious injuries with acute infections.
*m*) Wounds involving large vessels.
*n*) Recent hemorrhage.
*o*) Gassed cases.
*p*) Fractured spines.
*q*) All bad joint injuries.

Q. — 7. **What is the comparative condition of wounds arriving at the Base dressed with :**

*a*) Dry gauze.
*b*) Dichloramine-T.

*c*) A protective.

*d*) Rubber tubes.

*e*) Carrel-Dakin.

*f*) Vaseline gauze.

*g*) Bipp.

*h*) Flavine.

A. — Votes expressing best results: Dry gauze, 13. Carrel-Dakin, 12. Vaseline gauze, 5. Flavine, 3. Dichloramine-T, 1. Rubber tube, 1. Protective, 1. Bipp, 0.

Remark: Nearly all observers emphasize the risk of packing the wound *tightly* with any dressing — lightly placed surface dressings necessary.

A thorough primary operation procedure associated with proper splinting much more important than any type of dressing used.

II. Gas gangrene.

Q. — 1. **To what extent, if at all, do the following predispose to gas gangrene?**

*a*) Ligation of main artery of a limb.
*b*) Tight bandages.
*c*) Tight packing of a wound.
*d*) Insufficient debridement.
*e*) Low vitality from shock and hemorrhage.

A. — General concensus that all factors mentioned predispose as follows: —

*d*) First cause.
*a*) Second cause.
*b-c*) Third cause.
*e*) Fourth cause.

Q. — 2. **What is the indication for local operation? For amputation?**

A. — Agreement that essential indication for local operation is: — involvement of definite muscle or small muscle group which can be completely removed without loss of function of limb. For amputation — evidence of massive gangrene of limb; where removal of involved tissue cannot be accomplished without destruction of function of limb; when complicated by serious injury to main arteries of limb; when extensive fracture of large bones and joints co-exists; in doubtful cases in which patient suffers from extreme shock and hemorrhage; and where there are other wounds requiring operation in which there is general constitutional evidence of a severe fulminating type of infection.

Q. — 3. **What is the value of anti-gas sera?**

A. — Base Hospital No. 19. 15 cases with sera treatment previous to admittance : 4 of these developed gas infection, 2 died, and 2 recovered, the 2 dying requiring additional operation after admittance.

Base Hospital No. 48. Enthusiastic over serum as prophylactic — Bull's serum, 40 cc. intravenous, repeated in 30 cc. dose, with rising pulse, the open wound being washed with hydrogen peroxide.

One response. " Nil ".

Base Hospital No. 6. " Favors for both prophylaxis and early treatment. "

A. R. C. Military No. 2. Experience limited. In few cases used — not encouraging. Trouble to get best anerobic sera. Experimental evidence good, and practical tests show no prophylactic or therapeutic value of the gas bacillus anti-toxin; other anerobic anti-sera in hands of certain French physicians show evidence of possible value.

Q. — 4. **Is it justifiable to base local operation or amputation on the bacteriological findings alone?**

A. — " No " sums up answer to this question.

Q. — 5. **Is the general range of temperature high or low? Pulse rate high or low?**

A. — General agreement that pulse runs high and temperature comparatively low — pulse from 110-130 with temperature 101-102°F. in average cases.

Q. — 6. **How frequently does gas gangrene attack tissues other than muscle?**

A. — Responses : —

*a*) Secondary involvement of subcutaneous tissue.
*b*) Chest involvement not uncommon.
*c*) Often begins in ecchymoses of fascia and in blood clots.
*d*) In many autopsies gas found in liver.

Remark: All other reports agree that tissue other than muscle is rarely affected. The above remarks are additional statements from four hospitals.

III. Debridement.

Q. — 1. **What is included in a good debridement?**

A. — Concensus: —

*a*) Sparing removal of skin about wound margin.
*b*) Any necessary enlargement of wound and proper retraction.
*c*) Removal by sharp dissection of all contaminated, contused, devitalized tissue lining wall of tract of missile, with avoidance of injury of any important blood vessels and nerves.
*d*) Removal of foreign bodies including pieces of clothing.
*e*) Scrupulous hemostasis, and drying by ether lavage (optional).
*f*) Fixation of remaining organisms by tincture of iodine (optional, but believed by some to be of value).
*g*) Especial care with regard to muscle tissue removed, with contraction, bleeding and normal muscle color, the guides in reaching normal muscle tissue.
*h*) Guarded removal of unattached bone fragments.
*i*) Direct observation, by good head-light, of wound tract.

Base Hospital No. 48 emphasizes great importance of transfixion suture (pig's-gate) in depths of posterior-tibial or similar wounds, believing it to save much secondary hemorrhage and many lives.

Q. — 2. **What errors in debridement have you noted?**

A. — Concensus: —

*a*) Inadequate exposure of wound tract.
*b*) Incomplete removal of damaged and contaminated tissue.
*c*) Unnecessary transverse section of muscle.
*d*) Undue sacrifice of skin (important).
*e*) Too firm packing with gauze and through-and-through drainage.
*f*) Unnecessary damage to important vessels and nerves.
*g*) Short cuts to foreign bodies without following wound tract.

IV. Tetanus.

Q. — 1. **How many cases?**

A. — Cases seen. — 91. To total admissions — .018 %.

Q. — 2. **Results?**

A. — 37 deaths. Rate — 61.66 %.

Note. — Death percentage based on 60 cases with complete data. 31 cases from one hospital not giving deaths not included in this percentage.

Q. — 3. **Are there any contra-indications to giving a second dose of anti-serum?**

A. — Opinions : —

*a*) Anaphylactic reaction following first dose.
*b*) If an anaphylactic individual is properly desensitized, no objection.
*c*) Unwise if full dose has been given and patient is profoundly septic.
*d*) No, if given before ten days.
*e*) Yes — severe and dangerous primary reaction.

Remark : With exception of above opinions, all observers agreed that there was no objection to a second dose.

Q. — 4. **Have you seen local tetanus?**

A. — Votes. — Yes — 7.
No — 27.

Q. — 5. **Discuss late tetanus — cause — prevention.**

A. — Expressions of cause : —
*a*) Re-opening of old wound in operation around rectum.
*b*) Mechanical trauma — operative or other.
*c*) Insufficient primary operation; secondary operation; insufficient drainage; improper dressing; insufficient serum.

Prevention : Correction of above and always giving serum before secondary operation.

Remark : Most hospitals stated that they had seen little or no late tetanus.

V. Delayed primary closure.

Q. — 1. **Bacteriological control or clinical judgment?**

A. — Votes

- Bacteriological control — 5. (Base 6 laboratory has once given 168 reports in 48 hours).

- Clinical judgment — 10.

- Both when possible — all.

Q. — 2. **Average time after primary operation?**
3. **Percentage of successes?**
4. **Has there been loss of life or limb following failures?**

| | 2. | 3. | 4. |
|---|---|---|---|
| A. — | 10-12 days | 98 o/o | 1 (death tetanus). |
| | 10 " | 65 o/o | o |
| | Undetermined | 94 o/o | o |
| | 3 days | 95 o/o (about) | o |
| | 2-5 " | — | o |
| | 3-4 " | 80 o/o | 2 (deaths gas infect). |
| | 3 " | 95 o/o | o |
| | 10 " | 75 o/o | o |
| | 10 " | 85.9 o/o | o |
| | 3-5 " | 90 o/o | o |
| | 4-7 " | 90 o/o | o |
| | 10 " | 95 o/o | o |
| | 6 " | 65 o/o | o |
| | 2-4 " | 70 o/o | o |
| | 8 " | 75 o/o | 1 (limb). |

Remark : It is noted that the first hospital, Base No. 6, relied, with special care, on bacteriological findings by culture methods.

VI. Pre-operative cases.

Q. — 1. **What types of case need no operation?**

A. — *a*) Perforating bullet wounds (including machine-gun and rifle) of soft parts, *exclusive* of belly wounds, with no marked hematoma or other evidence of marked injury to important vessels, and with no evidence of serious nerve lesions; with small wound of entrance and exit, and without marked bone injury, provided there is no obvious infection present.

*b*) Minute foreign bodies in small penetrating wounds.

*c*) Non-sucking wounds (penetrating and perforating chest) without symptoms; or early cases of the same type without serious internal or external hemorrhage.

*d*) Certain head cases — extreme care in determining.

Q. — 2. **How have cases evacuated without operation (pre-operative) done?**

A. — Ten reports — no experiences.

*Well.*

*a*) If within 24 hours; wounds of buttocks.

*b*) Perforating, except belly and head.

*c*) Slight superficial.

*d*) All.

*e*) All.

*f*) All.

*g*) All.

*h*) Superficial.

*i*) All.

*j*) All.

*k*) Except tarsal involvement.

*l*) All.

*m*) All simple perforated of chest and soft parts.

*n*) Majority.

*o*) All except calf-buttock, thigh, thorax and subscapular.

*p*) All.

*Badly.*

*a*) Majority infected.

*b*) All serious wounds.

*c*) None as well as operated cases.

*d*) Infection except in perforating wounds.

*e*) All.

*f*) 95 o/o badly.

*g*) All.

*h*) Higher mortality.

*i*) All except simple perforating of soft parts and chest.

Q. — 3. **List the types of cases suitable for evacuation without operation.**

A. — See Heading No. 1. Many wounds of hands and feet, a few spine, and some head cases.

Q. — 4. **What are the advantages and the disadvantages of the pre-operative train?**

A. — 10 reports — no experience.
All others agree that properly scheduled trains clear front area hospitals, in time of stress, of cases in which operation can be delayed without danger to life or limb or function.

VII. Chest surgery.

Q. — 1. **What are the indications for operation in the front areea?**

A. — *a*) Sucking chest wounds.
*b*) Serious hemorrhage.
*c*) Large effusions.
*d*) Driven-in bone fragments.
*e*) Large foreign bodies.
*f*) Collapsed lung — same side as wound.
*g*) Much rib comminution.
*h*) Infection.
*i*) Hemothorax with tension.
*j*) Pneumothorax with tension.
*k*) Foreign body in heart or pericardium.
*l*) Foreign body in mediastinum.
*m*) Simultaneous wounds — both sides of chest.
*n*) Hemothorax plus anerobic infection.

Q. — 2. **What are the indications for operation in the Base?**

A. — *a*) Empyema.
*b*) Lung abscess.
*c*) Secondary hemorrhage.
*d*) Sinuses due to foreign bodies.
*e*) Pus pockets.
*f*) Hemothorax with symptoms. (Repeated aspirations when necessary.)
*g*) Radical thoracotomy for infected hemothorax.

Q. — 3. **Discuss the anesthesia — the operation technic.**

A. — Anesthesia. Local, if possible, recommended by all.

| | | |
|---|---|---|
| Stated first choice. | Gas Oxygen | 18 |
| | Ether | 9 |
| | Chloroform | 2 |
| | With morphine and atropine | 3 |
| | Warm ether | 1 |

Operative technic. Definite opinions : — For hemorrhage and removal foreign bodies : 6″ incision, 5″ of 4th rib removed. Begin incision at costal cartilage. Rib spreading. Split fibers pectoralis major. Lung delivered through wound. Palpation. Incision into lung to deliver f. b. Lung sutured by fine catgut. Hot sponge for oozing. Fine catgut for other bleeding. Gentle sponging of blood from chest cavity. Parietal pleura closed by continuous fine suture — catgut. Muscle and skin sutured to make air-tight. All these chest wounds to be closed primarily.

Fluoroscopic table often used.

Always practice principle of thorough debridement and following of tract whenever possible in primary removal of foreign bodies — with radioscopic assistance.

Septic cases — thoracotomy; suction drainage, positive pressure by blow bottles as soon as possible.

Q. — 4. **Discuss the after-treatment.**

A. — *a*) Sitting posture.
*b*) Morphine as indicated.
*c*) Aspirations of effusions after 48 hours for drainage and displacements.
*d*) Prompt radical drainage for infections (optional carrelling of pleura when no open bronchus).
*e*) Cases should not be evacuated.
*f*) Rest essential.
*g*) Lungs expanded regularly as soon as condition permits. (James' bottle method satisfactory).
*h*) Warm and dry climate afterwards, if possible.

Remark : Eight expressions, only, in favor of Carrel-Dakin or other irrigation in infected chests. With these exceptions, general agreement in above principles.

VIII. Secondary Hemorrhage.

Q. — 1. **In what type of case does it usually occur?**

A. — Most commonly in extremities.

Concensus : —

a) Infected wounds with or without primary injury to vessels; usually primary injuries.
b) Infection after extensive debridement in presence of foreign bodies.
c) Prolonged use of drainage tubes.
d) Compound fractures with gas infection.
e) Following amputations.

Base Hospital No. 2, B. E. F. Series of 46 cases :

a) Comminuted fractures, tibias and fibulas, 17 o/o.

b) Fractures of humerus 13 o/o.

c) Wounds of thigh, without fracture, 13 o/o.

d) Buttock wounds 11 o/o.

e) Wounds popliteal region 11 o/o.

Q. — 2. **Should first indication be ligation or temporizing?**

A. — Stated preference. For ligation, 24. For temporizing, 3. A. R. C. Military Hospital No. 2. " Ligation, if can be done in wound. Hesitate before ligating an arterial trunk to stop hemorrhage in an infected wound. Amputation often preferable, especially when complicated by gas. "

Q. — 3. **What are the predisposing causes of secondary hemorrhage?**

A. — a) Improper hemostasis.
b) Early transportation of amputations and hemophilics.
c) Faulty debridement.
d) Insufficient drainage.
e) Excessive restlessness.
f) Traumatic aneurism.
g) Rubber tubes in proximity to vessels.
h) Faulty ligatures.

Remark : One definite expression that Carrel-Dakin solution favors hemorrhage, by dissolving ligatures and loosening blood clots.

Q. — 4. **What is the general treatment?**

A. — General agreement :

a) Transfusion of blood after control of hemorrhage.
b) Fluids by mouth and rectum or subcutaneously, forced.
c) Absolute quiet.

*d*) All general treatment of shock.
*e*) Intravenous saline inferior to transfusion.

IX. Knee-joints.

Q. — 1. **In through-and-through machine gun or rifle wounds, how do non-operative compare with operative results?**

A. — Definite opinions : Non-operated cases : —

| | | |
|---|---|---|
| | " Do as well as " | 9 |
| | " Not so well " | 4 |
| | " Do better " | 17 |
| Most qualify by remark, | " Unless extensive bone injury ". | |

Q. — 2. **In debridement do you advise :** —
*a*) Complete closure?
*b*) Closure of capsule and fascia?
*c*) Leaving the wound entirely open?

A. — Votes : — *a*) 15.

*b*) 20.

*c*) 1.

Seton drainage to capsule recommended by two.

Q. — 3. **Is shattering the head of the tibia or of the condyles of the femur the more serious?**

A. — Votes : —

| | | |
|---|---|---|
| Head of Tibia. . | 17. |
| Equally serious . | 4. |
| Condyles . . . . | 10. |

Base Hospital No. 2, B. E. F. " Injured condyles with joint-involvement, 40. Infected, 19. Amputations, 18. Head tibia with joint-involvement, 15. Infected, 7. Amputations, 5. "

Q. — 4. **What type of knee-injury demands immediate amputation?**

A. — Consensus : —

*a*) Extensive destruction both bones beyond limits of functional recovery, with associated injury to main vessels.
*b*) Extreme destruction with fulminating infection.
*c*) With extensive comminution of lower third of femur or upper third of both bones of leg, or when popliteal artery is severed.

Q. — 5. **What type of infection and what extent of involvement of the joint demands amputation?**

A. — Concensus : Streptococcus infection of fulminating type with severe systemic symptoms, progressive, especially when associated with much bone injury, or when infection extends to muscle-planes, or when burrowing abscesses form. The gas bacillus and staphylococcus not as frequent agents as the streptococcus in necessitating amputations.

Base Hospital No. 2, B. E. F. " In general, panarthritis demands amputation, though resection is sometimes possible in specially favorable cases. "

Their figures are : —

| | Cases. | Amputations. |
|---|---|---|
| Streptococcus . . | 11 | 9 |
| Staphylococcus. . | 6 | 3 |
| B. Welchii. . . . | 7 | 5 |

Q. — 6. **In knee-joint injury and infection, has more error been made in conserving or in amputating?**

A. — Votes : Conserving . . . 21.
Amputating. . . 5.

Remark : One expression that error has been in amputating too frequently at the front and conserving too much at base.

Q. — 7. **What effect has excision of the patella on the function of the joint?**

A. — Most observers agree that excision results in marked interference with function, the joint being weakened to such an extent that mechanical apparatus is necessary; that the patellar ligament serves, with difficulty, the function of extension unless the knee is thrown in backward curve. Stiff joint also stated to occur.

Definitive Votes:

- Good function. . . 3.

- Poor function . . . 9.

- Little effect . . . . 3.

Q. — 8. **Compare mobilization with immobilization in the treatment of joints (Willem's method).**

A. — Votes. For immobilization. . . 6.
For mobilization . . . . 16.
Remainder either no expression or misinterpreted question.

Q. — 9. **What is your estimate of the value of antiseptic treatment of knee-joints?**

A. — Votes : Desirable . . 7. (Carrel-Dakin used by these.)
Undesirable. 25.

X. Antiseptics.

Q. — 1. **Compare the principle of chemical antiseptics with the principle of non-chemical treatment of infected wounds.**

A. — Treatment by chemical antiseptics preferred by 28.
Non-chemical treatment preferred by . . . . . 7.

Remark : Carrel-Dakin method choice among those using antiseptics.

Q. — 2. **List antiseptics in order of their availability for battle conditions at the front areas — at the Base.**

A. — Availability Order : —

| *At Front.* | *At Base.* |
|---|---|
| Iodine. | Carrel-Dakin. |
| Carrel-Dakin. | Alcohol. |
| Alcohol. | Iodine. |
| Flavine. | Dichloramine-T. |
| Mercury Salts. | Mercury Salts. |
| Ether. | Boric-Acid. |
| Dichloramine-T. | Ether. |
| Lysol. | Picric Acid. |
| Phenol. | Lysol. |
| Bipp. | Bipp. |
| Acetic Acid. | Acetic Acid. |
| Balsam Peru and | Burrow's Solution. |
| Castor Oil. | Iodoform. |

XI. Anesthetics.

Q. — 1. **How do you value the nurse anesthetist?**

A. — Votes
- Very satisfactory. 25
- Satisfactory . . . 8
- Unsatisfactory. . 1 (For prolonged operations.)
- Corps men . . . 1
- None used. . . . 1

Q. — 2. **In what cases and under what circumstances may local anesthesia be used? Regional? Spinal?**

A. — Definite opinions :

*Local.*

1. Selected head cases.
2. Thoracotomy.
3. Dental Surgery.
4. Small surface operations with superficial foreign bodies.
5. Face operations.
6. Secondary closures.
7. All chest where general anesthetic contra-indicated.
8. Selected abdominal cases.
9. Majority spinal cases.
10. Drainage of abdomen if general anesthetic contra-indicated.
11. Many brain cases.
12. Superficial abscesses.

*Regional.*

1. Maxillo-facial surgery often.

2. Operations in orbit and about orbit.

3. Certain cases of skin-graft.

4. Certain spinal cases.

5. In clean surface operations, too extensive for simple local anesthetic, where general is contra-indicated.

*Spinal.*

1. Certain shock cases, combined with gas oxygen or with morphine plus hyoscin.
2. Crushed legs plus bladder injury, if not too low blood-pressure.
3. Amputations of lower extremities in desperate cases.
4. Perineal wounds where general anesthetic contra-indicated.

Q. — 3. **In what cases is gas and oxygen especially indicated?**

A. — Concensus : —

*a*) Chest cases frequently. (With acute bronchitis.)
*b*) With tuberculosis (pulmonary). (With gas complication.) (With influenza.)
*c*) Short debridement.
*d*) All cases except brain and abdominal, especially chest and shock.
*e*) Septic.
*f*) All lung operations.
One expression that ether is preferred in all cases.

Q. — 4. **Has the type of anesthetic influenced results?**

A. — Votes : Yes, 30. No, 3. One states " Not as important as anesthetist ".

Q. — 5. **How do you value Depage anesthesia?**

A. — 17 hospitals report " No experience ".
7 hospitals report " Highly valued ".
2 hospitals condemn.
1 hospital " No better than ethyl-chloride ".

Base Hospital No. 22. In 50 cases use very satisfactory.
Base Hospital No. 60. Most practical general anesthetic for front area.
Quick narcosis and recovery. Easily transported. Used in over 200 cases.

XII. Fluids:

Q. — 1. **Compare sodium bicarbonate with normal saline in treatment of shock and hemorrhage.**

A. — Votes

| | |
|---|---|
| Normal saline superior | 11 |
| Sodium bicarbonate superior | 4 |
| No difference | 10 |
| No opinion | 8 |
| Both non-essential | 2 |

Q. — 2. **Compare intravenous saline infusions with giving water by mouth or rectum, or subcutaneously.**

A. — Concensus: That whenever possible water should be given by mouth and rectum, as by the mucous membrane it is almost entirely absorbed, though comparatively slowly. A few prefer intravenous under all conditions, but most state a preference for its use when quick action only is desired after acute hemorrhage, or in shock, using subcutaneous method when less haste is desired.

Q. — 3. **Compare gum-salt with salines; with blood.**

A. — Votes:

| | | |
|---|---|---|
| 1st place | blood | 31 |
| | gum-salt | 0 |
| | saline | 0 |
| 2nd place | blood | 0 |
| | gum-salt | 13 |
| | saline | 10 |
| 3rd place | blood | 0 |
| | gum-salt | 9 |
| | saline | 10 |

Q. — 4. **Have you noted any ill effects from gum-salt?**

A. — Votes : Yes, 13. No, 11.

Base Hospital No. 20. Unfavorable reaction in 15 o/o of the cases. 3 deaths attributed to solution.

Base Hospital No. 6. Nothing to recommend it.

Base Hospital No. 19. 2 cases.

Base Hospital No. 15. Considered dangerous.

Base Hospital No. 48. Several deaths attributed to its use. Very positive against its use.

XIII. BLOOD TRANSFUSION.

Q. — 1. **What method preferred?**

A. — Votes

| | | |
|---|---|---|
| A. — Votes | Sodium citrate . . . . . . . . . . | 26 |
| | Whole blood with paraffin tube. | 3 |
| | Kimpton-Brown method . . . . | 1 |
| | Syringe method . . . . . . . . | 1 |

Additional Expressions : 3 votes for citrate in front area. Indirect tube method for base, 1 vote. 1 states paraffin tubes and citrate equally successful.

Q. — 2. **Have there been any serum reactions when properly grouped?**

A. — Votes : No, 29. Yes, 0. Slight and rare, 5.

Q. — 3. **What results in prolonged infections?**

A. — 10 hospitals report " No improvement ".
14 hospitals report " Definite improvement ".
2 hospitals report " Temporary improvement ".
7 hospitals report " No experience ".

Q. — 4. **Discuss available sources, difficulties encountered, etc.**

A. — Sources.
*a*) Corps men.
*b*) Prisoners (carefully selected).
*c*) Slightly gassed and wounded, very carefully selected.

Difficulties
*a*) Length of time necessary to collect from donors.
*b*) Clotting in needle when injecting blood.
*c*) Inability to secure suitable donors.
*d*) Corps men off full duty from 24-48 hours after donation.
*e*) Keeping donors under careful control.

XIV. Amputations.

Q. — 1. **What is the value of the Guillotine operation?**

A. — Votes { Favorable. . . 19; Unfavorable. . 7; No advantage . 3; Speed only . . 7 }

Reasons for favorable votes { Rapidity. Drainage. Little shock. }

Q. — 2. **Is the medio-tarsal amputation justifiable?**

A. — Yes, 11. No, 18. No observation, 2. Non-committal, 3.

Q. — 3. **Compare the Symes' and the lower third amputation.**

A. — Votes : For Symes', 5. For lower third, 24. Remainder either no experience or no preference.

Q. — 4. **Is the rule that stumps of the lower extremities shall have no terminal scar a good one? On the upper extremities is the terminal scar always correct?**

A. — Votes : *a*) Yes, 15. No, 8.

*b*) Yes, 11. No, 8.

Q. — 5. **Are amputations through the knee-joint recommended?**

A. — *a*) Yes, in emergency, 6. *b*) No, 28.

Q. — 6. **Shall the bones of the stumps be left with parallel or conical shape?**

A. — Votes : Parallel, 13. Conical, 8.

Q. — 7. **How near the knee-joint may amputations be made?**
8. **How near the elbow-joint?**
9. **Through the elbow-joint?**

A. — Concensus : In knee-joints a margin of from 3″ to 5″ above, and about 4″ or through tuberosities below, is the margin to be allowed; and just below the bicipital tuberosity and just above the condyles of the humerus is the margin for vicinity of elbow-joint. Amputations through the elbow-joint advised by only two.

XV. Head injuries.

Q. — 1. **Should all lacerations of the scalp be explored surgically for fracture even if fluoroscopic report is negative?**

A. — Votes : Yes, 31. No, 3.

Q. — 2. **Discuss foreign bodies in the brain.**

A. — Concensus : *a*) Accurate localization. *b*) Whenever accessible, foreign bodies should be removed unless multiple and minute, and through the tract of entrance, care being used to avoid further brain injury. Prolonged search with instruments or finger contra-indicated. Thorough cleansing of tract of softened tissue, bony fragments, dirt, etc., by catheter suction (gentle) and irrigation, then soft catheter as searcher. Foreign bodies in brain, with clean tract, not always a menace, and careful judgment should be exercised in their removal. Considered more important to clean the tract well than to remove foreign body, as abscess formation more frequent in tract than about foreign body.

Q. — 3. **Is the magnet useful in extracting large foreign bodies?**

A. — Votes : Yes, 12. No, 4.

Q. — 4. **Have you seen late abscesses?**

A. — Votes : Yes, 25. No, 4.

XVI. Hospital pboblems.

Q. — 1. **What improvement can you suggest in the arrangement of the standard American Base Hospitals?**

A. — Remark : Few opinions of definite nature.

Buildings more concentrated, especially surgical wards and X-ray room and laboratory; these preferably connected by corridors; special quarters for sick nurses; more liberal quarters for corps men — at present badly crowded; 1000 capacity instead of 500 would be more easily expanded; better facilities for heating receiving ward; larger and better equipped kitchens; better facilities for hot water; usually impractical to have special hospitals, but special services in Base centers should be arranged when distribution of cases is possible, with well-balanced staff to serve them.

Personnel : 250 corps men, at least; Sanitary Corps should handle all mess, Q. M., and supply problems; more permanent personnel, as constantly changing personnel interferes badly; more care in detaching personnel, thus preventing crippling of heavy services; competent permanent skeleton staff, with chief and assistant for each service.

Q. — 2. **What are the advantages and limitations of the Surgical Team?**

A.

Advantages.

*a*) Marked : " Rank should not remove experienced surgeon from the wounded soldier ".

*b*) Elastic and mobile service for reinforcement.

*c*) Complete independent mobile unit.

Limitations.

*a*) Too small.

*b*) Should be organized alway according to surgical ability and not rank.

Q. — 3. **What should be the size of a Mobile Hospital?**

A. — *a*) About 200 beds with accessory hospitalization equipment.

*b*) 300-600 beds : in Bessoneaux — 10 teams, 5 day, 5 night; 150-200 major operations in 24 hours.

Remark : Few answers — most stated no opinion.

Q. — 4. **What should be the equipment of a Mobile Hospital?**

A. — *a*) Such as to enable it to operate independently.

*b*) About same as French auto-chir; with American instruments and X-ray apparatus.

Remark : Few answers and no statements of complete equipment.

Q. — 5. **What should be the function of Mobile Hospitals?**

A. — Concensus : —

*a*) Must be *very* mobile to serve any other purpose than the usual Evacuation Hospital serves.

*b*) Must advance with army to handle all urgent cases, and evacuate as early as possible. Located as near front lines as particular sector conditions permit.

Q. — 6. **Is it best to have special hospitals, such as head, chests, fractures, or to have special wards for such cases in general hospitals?**

A. — Votes : For general hospitals . . . 30
For special hospitals . . . 5

Q. — 7. **What suggestions as to : —**

(1) Surgical instruments.

*a*) Quality. *b*) Types. *c*) Quantity.

(2) Dressings.

(3) Special types.

(4) Bandages.

*a*) Special. *b*) Splints.

A. — Concensus : —

(1) *a*) Quality, in general, should be higher.

*b*) Many missing types for special work.

*c*) Quantity recorded as sufficient in about one half of the responses.

(2) (3) (4) Dressings, bandages and splints abundant and satisfactory.

---

### SPECIMEN REPLIES TO QUESTIONNAIRE ON SURGERY AT THE BASE

---

### BASE HOSPITAL No 8.

I. General.

1. What is the total number of admissions to your hospital? 31,500.

2. What is the number of deaths? 186. How many deaths from pneumonia? 79.

3. What is the number of surgical cases? 19,579. Number of deaths? 46. Among these, how many were caused by pneumonia? 9.

4. How long should abdominal cases be held at the front before transportation? Very few abdominal cases reached here.

5. How do through-and-through chests travel? Very well.

6. What type of cases are most injured by travel? Head, abdominal and improperly splinted fractures, particularly of the lower limbs.

7. What is the comparative condition of wounds arriving at the Base dressed with :

| | |
|---|---|
| *a*) Dry Gauze. | Dry Gauze best. |
| *b*) Dichloramine-T. | No experience. |
| *c*) A Protective. | Not good. |
| *d*) Rubber Tubes. | Not good. |
| *e*) Carrel-Dakin. | Not good because poorly done in transit. |
| *f*) Vaseline Gauze. | Fair. |
| *g*) Bipp. | No experience. |
| *h*) Flavine. | No experience. |

II. Gas Gangrene.

1. To what extent, if at all, do the following predispose to gas gangrene?

| | |
|---|---|
| *a*) Ligation of main artery of a limb. | Predisposes to gangrene. |
| *b*) Tight Bandages. | Have never observed it. |
| *c*) Tight packing of wound. | Have never observed it. |
| *d*) Insufficient Debridement. | Probably most frequent predisposing. |
| *e*) Low vitality from shock and hemorrhage. | Not observed. |

2. What is the indication for local operation? Degree of toxemia, location, extent and nature of spread of infection. For amputation? The indication for amputation when localized, removal of infected area, will leave limb without function; in joints, destruction beyond limits of functional resection.

3. What is the value of anti-gas sera? No experience.

4. Is it justifiable to base local operation or amputation on the bacteriological findings alone? No.

5. Is the general range of temperature high or low? Temperature not characteristic. Pulse high or low? Pulse rate, the tendency to be high.

6. How frequently does gas gangrene attack tissues other than muscle? Rarely if ever.

*a*) Head, never observed.
*b*) Neck, very rarely.
*c*) Hands, rarely.

III. Debridement.

1. What is included in a good debridement? Removal of narrow skin margin and a long skin incision, cross slitting deep fissures to avoid strangulation of muscles and to permit draining. The removal of injured and infected muscle to prevent undue herniation and to prevent over-riding muscle planes from interfering with direct drainage. Great pains should be taken in handling and preservation of nerves. When bone is injured, the wound should be converted into a large funnel shaped cavity capable of draining itself, and in which free inspection and treatment of the bone is possible during dressing. Experience shows that it is impossible to judge at the primary operation of what fragments must be removed. Subperiosteal infected bone margin. The best rule is to remove detached fragment, but with the idea that the removal of other fragments should be done from time to time by the Base Surgeon.

2. What errors in debridement have you noted? The majority of cases with compound fractures reaching this Base Hospital are infected. The conclusion drawn is that there has been insufficient debridement. On the other hand, a certain number of cases have been observed with too extensive debridement with nerve injury. Some of these cases have been clean and it is therefore impossible to say that the operation was too radical. A very small proportion of the cases arriving at this hospital were in a condition to be secondarily closed.

IV. Tetanus.

1. How many cases? Three.

2. Results? One death.

3. Are there any contra-indications to giving a second dose of antiseptic serum? No.
4. Have you seen local tetanus? Not observed.
5. Prevention? Proper debridement and use of serum.

V. DELAYED PRIMARY CLOSURE OF WOUNDS.

1. Bacteriological control or clinical judgment? Qualitative bacteriological evidence more important than quantitative.
2. Average time after primary operation? Three to five days. Very few observations.
3. Percentage of successes? 90 percent.
4. Has there been loss of life or limb following failures? Neither observed. Several cases of septicemia have been observed.

VI. PRE-OPERATINE CASES.

1. What types of cases need no operations?
2. How have cases evacuated without operation (pre-operative) done?
3. List the types of cases suitable for evacuation without operation?
4. What are the advantages and the disadvantages of the pre-operative train?

The above refers to front area triage.

VII. CHEST SURGERY.

1. What are the indications for operation in the front area? Sucking wounds, uncontrollable hemorrhages and foreign bodies.
2. What are the indications for operation in the Base? Infected pleural contents, foreign body and abscess.
3. Discuss the anesthesia — the operation technic. Local, thoracotomy, straight ether for more extensive operation. Sub-periosteal rib resection for drainage and more extensive rib resection and decortication for others.
4. Discuss the after-treatment. Ordinary drainage without negative pressure, sitting position with intermittent installations of Dakin's solution for pyothorax. Our best results in empyema have been obtained by a resection of two ribs, removal of the fibrin, packing the cavity tightly with dry gauze, which is allowed to remain for 24 hours. It is then removed under a slight anesthesia. The wound is then lightly packed for three days with gauze which is removed every 24 hours.

At the end of the third day, drainage is installed. Irrigation is then begun. We have observed the same results from the use of Dakin, weak iodine solution and sterile water.

VIII. SECONDARY HEMORRHAGE.

1. In what type of cases does it commonly occur? Compound infected fractures of the femur, particularly when involving the lower third.
2. Should first indication be ligation or temporizing? Temporizing seldom, if ever, of any use.
3. What are the predisposing causes of secondary hemorrhage? *a*) Severe sepsis. *b*) Placing of rubber tubes in proximity of vessels.
4. What is the general treatment? Ligation, transfusion, and, usually later, amputations.

IX. Knee-joints.

1. In through-and-through machine gun or rifle wounds, how do non-operative compare with operative results? Non-operative cases are better than the operative cases.

2. In debridement, do you advise?

a) Complete closure? No.
b) Closure of capsule and fascia? Yes.
c) Leaving the wound entirely open? Leaving skin unsutured.

The large majority of post-operative cases which we have seen in this hospital have been infected.

3. Is shattering the head of the tibia or of the condyles of the femur the more serious? Injuries to the head of the tibia by far the more serious.

4. What type of knee injury demands immediate amputation? When general condition permits, immediate amputation is rarely indicated. There is no single type of knee injury which we have observed in this hospital.

5. What type of infection and what extent of involvement of the joint demands amputations?

Neither type of infection nor extent of involvement of knee joint, but the general condition of the patient, or complications.

6. In knee-joint injury and infection, has more error been made in conserving or in amputating? Greater error has been made in conserving.

7. What effect has excision of the patella on the function of the joint? The cases all too recent to permit of judgment.

8. Compare mobilization with immobilization in the treatment of joints. (Willem's method). The cases arriving here are too late for Willem's treatment.

9. What is your estimate of the value of antiseptic treatment of knee-joints? Non-essential.

X. Antiseptics.

1. Compare the principle of chemical antiseptics with the principle of non-chemical treatment of infected wounds. Other conditions being equal, chemical antiseptic offered material assistance.

2. List antiseptics in order of their availability for battle conditions at the front areas? At the Base? Impossible to answer.

XI. Anesthetics.

1. How do you value the nurse anesthetist? Highly.

2. In what cases and under what circumstances may local anesthesia be used? Regional? Spinal?

a) Clean minor cases.
b) Regional, not used.
c) Spinal, not used.

3. In what cases is gas and oxygen especially indicated? Severely septic cases; in very bad general conditions; shock and chest cases.

4. Has the type of anesthetic used, influenced results? Yes.

5. How do you value Depage anesthesia? Highly, in certain type of cases.

XII. Fluids.

1. Compare sodium bicarbonate with normal saline in treatment of shock and hemorrhage. Non-essential.

2. Compare intravenous saline infusions with giving water by mouth or rectum or subcutaneously. Non-essential.

3. Compare gum-salt with salines; with blood. Gum-salt does not compare with saline nor with blood.

4. Have you noted any ill effects from gum-salts? Yes, employed in two cases, both of which died in exactly the same manner and same length of time following its use.

XIII. Blood transfusion.

1. What method preferred? Citrate method.

2. Have there been any serum reactions when properly grouped? No.

3. What results in prolonged infections? No improvement noted.

4. Discuss available sources, difficulties encountered, etc. Plenty. Difficulties encountered, none.

XIV. Amputations.

1. What is the value of the Guillotine operation? Of value when combined with traction and during periods when impossible to carefully observe patients.

2. Is the medio-tarsal amputation justifiable? Yes.

3. Compare the Symes and the lower third amputation. Cases too recent to judge.

4. Is the rule that stumps of the lower extremities shall have no terminal scar, a good one? On the upper extremities, is the terminal scar always correct? Location of this scar unimportant. Upper extremity, terminal scar best.

5. Are amputations through the knee-joint recommended? No, except as preliminary to secondary amputation above condyle.

6. Shall the bones of all stumps be left with parallel or conical shape? Question impossible to interpret.

7. How near the knee-joint may amputations be made? Two inch stump below knee useful. Through femur, amputation should be three inches from knee-joint.

8. How near the elbow-joint? Forearm, two inches below biceps insertion. Arm, just above condyle.

9. Through the elbow-joint? No.

XV. Head injuries.

1. Should all lacerations of the scalp be explored surgically for fracture even if fluoroscopic report is negative? Depends on neurological examination.

2. Discuss foreign bodies in the brain. If no indication of symptoms is present, disregard presence of foreign body.

3. Is the magnet useful in extracting foreign bodies? No magnet at hand.

4. Have you seen late abscesses? Yes.

XVI. Hospital problems.

1. What improvement can you suggest in the arrangement of the standard American Base Hospital?

2. What are the advantages and limitations of the Surgical Team? Constitution of the Surgical Team is at a disadvantage in removing personnel and valuable surgical talent, particularly at times when the volume of work is to increase.

3. What should be the size of a Mobile Hospital? 100 hospital beds or 200 cot-beds with crisis expansion to 400 cot-beds by means of accessory hospitalization unit for the area.

4. What should be the equipment of a Mobile Hospital? After standard French auto-cherpean with American instrumentation and X-Ray apparatus.

5. What should be the function of the Mobile Hospitals? To be utilized for the treatment of the seriously, non-transportable wounded — head, chest, abdomen, shock, hemorrhage, grave fractures. Employ two mobile hospitals in same area, the rear mobile hospital to " leap-frog " the forward mobile hospital with each successive advance of the fighting line.

6. Is it best to have special hospital — such as, head, chests, fractures, or to have special wards for such cases in general hospitals? The segregation of cases is most important and may be obtained in the majority of conditions in special wards of general hospitals. In certain conditions in which the number of specialists and the number of cases is small, it would be more advantageous to segregate these in special hospitals.

7. What suggestions as to :

(1) Surgical Instruments;

*a*) Quality? Poor.
*b*) Types? Fair.
*c*) Quantity? Adequate.

(2) Dressings? Adequate.

(3) Special Types? Special types obtainable, best made in hospital.

(4) Bandages? Plentiful.

*a*) Special? Satisfactory.
*b*) Splints? Difficult to obtain.

---

### BASE HOSPITAL No. 15, A. E. F.

I. General.

1. About 30,000.

2. 422. The only complete records we have include the period from July 1st to December 14th, and show by autopsy 176 deaths from pneumonia.

3. About 15,000. Medical and surgical records are not separated in our files. From July 1st to December 14th there were 87 deaths among the surgical cases, of whom 25 had pneumonia.

4. Two weeks.

5. Exceedingly well.

6. Post-operative head, chest, and abdominal cases.

7. Dry gauze acts as a stopper in a wound which might just as well have been sutured primarily. Rubber tubes were excellent so long as the wound was not also stopped with gauze. Carrel-Dakin cases arrived with gauze dry and the tubes often plugged with secretion or pus. Vaseline gauze made an eminently satisfactory dressing. No cases treated with Bipp or Flavine arrived.

II. Gas Gangrene.

2. Local operation should be performed when the infection is local, that is, limited to a muscle or muscle group, to fascial planes or to subcutaneous tissue. Amputate when infection is too extensive for local removal or when toxemia is present.

3. Anti-gas sera have seemed a help, some few lives saved have been attributed to its use.

4. No.

5. The general range of temperature is low and of pulse high.

6. Between 15 o/o and 20 o/o of the time.

III. Debridement.

1. A good debridement consists in the removal of all destroyed tissue and foreign bodies compatible with saving nerves and important vessels, including all loose and many attached fragments of bone, in careful hemostasis.

2. Apparently a needless debridement of skin, too little of muscle, and none at all of bone. The last was a most glaring error.

IV. Tetanus.

1. Three cases.

2. All died, though having had serum. One had as many as 150 injections, being a case developing about 50 days after injury, a CCF of the femur with FB not removed, and complicated with long low grade suppuration.

3. We saw no contra-indications to a second dose of anti-tetanic serum. A large percentage had urticaria between the sixth and tenth days after injection.

4. We have seen one case of local tetanus with deep wound of both buttocks, having serum at once and again on arrival at the Base 4 days later, starting contraction of legs and buttocks on 5th day, and dying on the 6th without others signs. Tetanus B found.

V. Delayed Primary Closure of Wounds.

1. For the most part clinical judgment decided the question.

2. About 6 days.

3. 65 o/o success, 15 o/o partly successful.

4. No loss of life or limb from attempts at closure.

VI. Pre-Operative Cases.

1. Bullet and ball wounds of extremities not injuring important vessels, the minute to small shell fragment wounds, particularly when multiple, and through-and-through wounds of the chest.

2. Well in the majority of the cases.

3. The same as (1).

4. The advantage is to lessen the load on front units; the disadvantage is that unsuitable cases are evacuated.

VII. Chest Surgery.

1. The open chests, possibly the bleeding chests and those with large shell fragments in the lung, present indications at the front.

2. Empyema, FBs in the pleural cavity or close to the surface of the lung, except if the FBs be small, constant hemoptysis and persistent irritable cough with FBs in the lung, are the indications at the base.

3. Local anesthesia for empyema, and ether for other operations were the anesthesias used here.

4. Symptomatic, rest and quiet.

VIII. Secondary Hemorrhage.

1. In suppuration.
2. Temporizing is fatal. Ligate immediately.
3. Suppuration. Some of us think Dakin's fluid increases tendency to hemorrhage by dissolving ligatures and loosening blood clots.
4. Allay nervousness and produce quiet with morphine; give infusion of salt solution; after which, if necessary, transfusion.

IX. Knee-Joints.

1. Very satisfactorily.
2. If debridement has been satisfactory, close completely, otherwise close synovial membrane and capsules. Leave joint open and start motion if suppurating.
3. Shattering condyles of the femur is the more serious.
6. It is impossible at the Base to say an error was committed at the front in amputating. At the Base we erred more in conservation.
8. We believe in the Willem's method. It has given us the only pleasing results obtained here.
9. In already suppurating knee-joints antiseptic treatment is of no avail.

X. Antiseptics.

1. Surgical procedure, as thorough debridement, with proper drainage is essential, and then chemical antiseptics are aids. The latter without the former are valueless.

XI. Anesthetics.

1. We have used the nurse anesthetist almost entirely and with satisfaction.
2. We never used regional or spinal anesthesia. Local anesthesia was used as always for abscesses and surface FBs, and often for craniotomy.
4. Local anesthesia bettered results in cra nial injuries.
5. The Depage method has been satisfactory with no bad effect.

XII. Fluids.

4. Gum-salt is considered dangerous, though it has not had much of a trial.

XIII. Blood Transfusion.

1. The citrate method is preferred.
2. No.
3. It is considered helpful.

XIV. Amputation.

1. We see no indication for the Guillotine operation, it always means re-amputation.
4. Weight bearing stumps should not have terminal scars.
5. An amputation might be done through the joint for suppuration, with a re-amputation after sterilization of the wound.
6. Weight bearing stumps should be squared off, not conical.
7. About 4 inches above or below.
8. Below the tuberosity of the radius.
9. There is no objection to amputation through the elbow-joint.

XV. Head Injuries.

1. Yes.

2. All head cases, even walking, should be explored with a free excision o the tract down to bone under local anesthesia. When bone penetration is present a block removal of bone when possible. With dural and brain penetration, the wound is gently irrigated and suction applied by a Dakin's syringe attached to a soft rubber catheter (the preferable irrigation is hot normal salt solution). Coughing by patient frequently helps by expelling bone fragments or even superficial FBs, all bone fragment and FBs should be removed when same is possible without further injury to brain substance. No actual brain or dural debridement is permissible because of possible resulting hemorrhage. Closure or drainage depends on condition of resulting wound. When drainage is necessary soft rubber tissue is most commonly used.

3. Yes.

4. Yes.

---

### BASE HOSPITAL No 12, U. S. A.

I. General.

1. Admissions :

| | |
|---|---|
| July 1, 1917 to January 1, 1918 | 19,861 |
| January 1, 1918 to December 1, 1918 | 35,740 |
| Total | 55,601 |

Daily average : 107.

2. Deaths :

| | | |
|---|---|---|
| July 1, 1917 to January 1, 1918 | | 143 |
| Due to pneumonia | 1 | |
| Gas cases | 22 | |
| January 1, 1918 to December 1, 1918 | | 261 |
| Sick cases | 103 | |
| Wounded | 158 | |
| Pneumonia | 67 | |
| Total | | 404 |

3. Number of Wounded :

| | |
|---|---|
| July 1, 1917 to January 1, 1918 | 11,691 |
| January 1, 1918 to December 1, 1918 | 17,067 |
| Total | 28,758 |
| Deaths . . . 1917 | 137 |
| 1918 | 158 |
| Total | 295 |

Pneumonia deaths : No post-operative.

Surgical sick not included in these figures.

4. Ideally, abdominal cases should be held until convalescent.

5. Through-and-through chest cases (bullet) travel very well. Shrapnel wounds travel poorly.

6. Chest, abdomen and head cases are most injured by travel in order named. Extremity cases travel best early, if well splinted.

7. Most important factor in the condition of wounds arriving at the base is careful complete excision at the front. Majority favor dry gauze. One man prefers flavine.

II. Gas Gangrene.

1. Most important is early excision of damaged tissue and removal of foreign bodies and cloth by wide incision. Avoid leaving blood clot.

The other factors mentioned are all predisposing but of lesser importance.

2. Local operation is indicated in early cases, limited to a muscle or muscle group, the removal of which will not leave a useless limb, otherwise amputation.

3. No data.

4. No.

5. Temperarure low — pulse high.

6. Chest involvement not uncommon.

III. Debridement.

1. Wide longitudinal incision to allow complete exposure and excision of all damaged tissue under sight.

2. Errors noted include :

*a*) Too wide skin excision, without cleaning the depth of the wound.

*b*) Insufficient exposure, and attempt to operate through small cone-shaped incision. Leaving cloth or foreign body in wound.

*c*) Bone injuries incompletely cleaned.

IV. Tetanus.

1. Two cases.

2. Both fatal.

3. B. E. F. gave two to four doses. Almost always two and usually more.

4. No.

5. Have seen only one case of recurrent tetanus. Undoubed tetanus in October, 1917, cured in hospital in England. Recurrence of symptoms and cure under subcutaneous injections of serum in March 1918. Foreign body and cloth removed from wound. Subsequent history not known.

V. Delayed Primary Closure of Wounds.

1. Clinical judgment.

2. 2-4 days.

3. 70 o/o complete success in 100 cases.

4. No.

VI. Pre-operative Cases.

1. *a*) Clean through-and-through bullet wounds without signs of hemorrhage or extensive bone injury.

*b*) Slight superficial wounds.

*c*) Small deep foreign bodies not infected or causing symptoms.

*d*) Many gutter wounds.

2.

3. See under (1).

4. No experience.

VII. Chest Surgery.

1. Sucking wounds, extensive rib comminution, septic wounds, troublesome hemothorax. Large fragments of shrapnel retained.

2. Usual indication for operation at the base is sepsis.

3. Local anesthetic preferred.

4. Repeated respiration for hemothorax and recurrence of fluids with bacteriological examination. Opiates and general care.

VIII. Secondary Hemorrhage.

1. Infected wounds. We believe there is usually a primary injury to the vessel, the hemorrhage being arrested by clotting. The clot is then removed by the infective process and hemorrhage takes place. We have frequently seen such vessels as the radial, ulnar, and profunda femoris completely divided.

2. Always ligation, preceded in extreme cases by transfusion.

3. Injury to vessel either opening into the lumen, with clotting, or injury to the wall. Always infection. Loss of tissue about the vessel. Pressure of bone fragments, or drainage tubes.

4. Blood transfusion, fluids, warmth, full diet. Rest with morphine if necessary.

IX. Knee-Joints.

1. Well.

2. Advise always closure of capsule. If the case is to travel at once the other tissues may be left open for delayed primary suture.

3. Shattering of head of tibia.

4. *a*) Extensive damage of both bones where capsule cannot be closed or where too much bone is gone for resection.

*b*) Extensive bone injury with injury of vessels or nerve or both.

5. Individual cases vary. In general, streptococcus infections with bone damage, or extensive shattering with whole joint infected, indicate amputation. Involvement of the subcrural pouch only is the most favorable form of infection.

6. Hard to state. In many doubtful cases much visceral damage has been done by conservation. We have in no case lost life by waiting, and have had no deaths from amputation in infected knee-joints not associated with other wounds.

7. No personal data.

8. Very little experience with mobilization. Believe infected joints should be immobilized.

9. No data.

X. Antiseptics.

1. Favor non-chemical.

2. Iodine, alcohol, picric acid at front. Have used iodine for preparation at the base.

XI. Anesthetics.

1. Nurse anesthetist is invaluable.

2. Local and regional where the condition of the patient contra-indicates general, especially heads and chests. In other cases general. Rush of work makes it impossible to use local in many suitable cases. Have used spinal in a few cases for amputation in desperate cases.

3. In the hands of an expert, gas and oxygen is the choice in nearly all cases.

4. Yes. Believe the extensive use of chloroform at the front as well as morphine-scopolamine has had injurious effects.

5. No experience.

XII. FLUIDS.

1. No data.
2. Have not used intravenous saline.
3. Gum-salt better than salines; inferior to blood.
4. No.

XII. BLOOD TRANSFUSION.

1. Whole blood — we have used the paraffin tube.

2. No.

3. Marked temporary improvement in most cases. We believe a number of cases have been saved.

4. We have used slightly wounded as donors, grouped a large number at a time. Have used paraffin tube method almost entirely and have had only a few difficulties due to imperfect technic.

XIV. AMPUTATIONS.

1. Guillotine amputation in urgent cases has advantage of rapidity and good drainage. Used where secondary operation is necessary. At the base we rarely used it.

2.
3. } No data as to end results.
4.
5. No.
6. Tibia conical.
7.
8. } No data as to end results.
9.

XV. HEAD INJURIES.

1. All infected scalp wounds should be cleaned and explored. Fluoroscope of no value as to fracture. We rely on symptoms and stereoscopic picture.

2.

3. In the minority of cases we have found magnet of value.

4. Yes.

XVI. HOSPITAL PROBLEMS.

1.
2.
3.
4.
5.
6. In general, special wards in general hospitals. In centers some special hospitals.
7.

### BASE HOSPITAL N° 6.

I. General.

1. Total number of admissions to this hospital : 23,288.

2. Number of deaths : 420. Deaths from pneumonia : 284.

3. Total number of surgical cases : 17,466. Number of deaths (entire service) : 77. Number of deaths (operated cases) : 52.

4. Abdominal cases should be held at the front before transportation up to 12 days if possible.

5. Through-and-through chest wounds travel with little inconvenience, those having an open sucking wound and septic cases would best be held at the front.

6. The cases most injured by travel are compound fractures of the femur, knees, and head cases.

7. Wounds arriving at the Base were usually in the following condition when dressed respectively with :

*a*) Dry gauze; in clean wounds the gauze adhered, in septic wounds the gauze acted as a stopper of wound discharges. Packing wounds with gauze would seem to have made the operator careless with his hemostasis.

*b*) Dichloramine-T; no wounds received dressed with dichloramine-T.

*c*) A protective; wounds left wide open and dressed with a simple protective give better results than when packed with gauze.

*d*) Rubber tubes; there has been no advantage of drainage of wide open wounds with rubber tubes, and there has been some disadvantage in the erosion of vessels from pressure.

*e*) Carrel-Dakin; our cleanest wounds were those amply filled with Carrel tubes and to which Dakin solution had been administered en route.

*f*) Vaseline gauze; vaseline gauze has been even worse than dry gauze. Its power of absorption of wound secretions and its draining power have been entirely lost and the wounds gummed with vaseline have been more septic.

*g* and *h*) We have seen no wound dressed with Bipp or Flavine.

II. Gas Gangrene (17 cases, 3 deaths).

1. All of the following factors predispose more or less to gas gangrene in the following order : *a*) Ligation of the main artery of the limb; *b*) Insufficient debridement and tight packing of wound; *c*) Tight bandages; *d*) Low vitality from shock and hemorrhage.

2. Local operation is indicated for the drainage of pent-up secretions and amputation only when the circulation below the point of injury is considerably interfered with, and when the life of the patient is at stake from a severe general toxemia. We have been very conservative in our local operations and amputations even in these cases of gas gangrene, and have usually done a minimum of manipulation after the administration of the Bull-Pritchett anti-gas serum.

3. We consider anti-gas sera of great value both as a prophylactic measure and in early treatment, and have administered it in all cases where the clinical appearance of the wound and bacteriological findings have enabled us to make a diagnosis of either gas gangrene or gas bacillus infection.

4. It is certainly not justifiable to base local operations or amputation on bacteriological findings alone. One must distinguish gas gangrene, mixed gas bacillus infection, and the presence of the gas bacillus in the wound with no clinical evidence of its presence.

5. The general range of temperature and pulse in gas gangrene is high temperature and moderately fast pulse at first, followed by moderately high or low temperature and very fast pulse. Following this sequence a fast pulse is a bad sign.

6. Gas gangrene attacks muscle with the greatest avidity but it may attack any soft tissue beneath the skin.

III. Debridement.

1. A good debridement may be expressed as a wide opening and the removal of all devitalized tissue to the very depth of the wound.

2. The principal errors in debridement have been cutting across important structure, waste of skin covering, not carrying the incision to the extreme depth and perpendicular with the skin, and insufficient removal of bone fragments.

IV. Tetanus.

1. Five cases, 4 deaths.

2. Results, miserable.

3. With the exception of the serum reaction, which has little or no lethal importance, there is no objection to the giving of a second dose of anti-tetanic serum, and certainly no objection after a period of from seven to ten days.

4. We have seen the tetanus bacillus from wounds without local or general symptoms from this bacterium.

5. We have had but one case of tetanus to follow a secondary and late manipulation of a wound and, in this case, three separate doses of anti-tetanic serum had been given, the last dose less than ten days before operation. It has been our rule to administer anti-tetanic serum in all cases of wounds coming to operation later than ten days after the last dose of serum. Herein lies the only means of prevention that we know of. Out of five cases where we were able to make a diagnosis there was no benefit gained from treatment.

V. Delayed Primary Closure of Wound.

1. Our secondary wound closures were controlled almost entirely by bacteriological findings. Smears were made on swabs and placed in culture tubes and sent to the Laboratory. These swabs were shaken off in serum broth, incubated for 24 hours, smeared on a slide and stained by Gram. The streptococcus and gas bacillus were detected microscopically by the fact that they retained Gram stain, by their manner of growth and their morphology. The Laboratory reported on as many as a hundred and sixty-eight such cultures in forty-eight hours. We have entirely disregarded quantitative bacteriological examination of wounds as prescribed by Alexis Carrel, and have depended entirely on these qualitative cultures. All wounds not showing streptococcus and gas bacillus, and which have had sufficient anti-tetanic serum within ten days (at least two doses), were closed immediately by our technic of secondary wound closures. This technic removes mechanically the bulk of all bacteria; when the streptococcus is found present the wound is treated with Dakin solution by the Carrel method until the streptococcus appears no longer in the cultures. When the gas bacillus was found the wound was similarly treated, and Bull-Pritchett gas bacillus antitoxin was given. The wound when very moist and covered with necrotic tissue was frequently chemically cleaned with Dakin solution.

2. Our effort was always to close wounds before the 12th day, before the epithelial edge was laid down and the skin too much retracted. We considered of importance a supple elastic skin, a debridement en masse so as to mechanically remove the bulk of all bacteria, a sufficient undermining of the skin that it might be readily closed without tension, and a careful hemostasis. In this way we have closed more than a thousand wounds.

3. We have 98 o/o healing per primum or a gain of at least 85 o/o surface closure. Approximately 75 o/o of these cases were returned to duty.

4. Only in one case have we had a loss of either life or limb, and this was a case of tetanus following a secondary wound closure, though the case had received three doses of anti-tetanic serum and the last dose less than ten days before the operation.

VI. Pre-operative Cases.

1. The types of cases not needing operation are perforating and penetrating, usually perforating, rifle and machine gun wounds, with the exception of the abdomen.

2. Such cases with few exceptions and even those with fracture have done remarkably well.

3. Perforating rifle and machine gun wounds of the soft parts and even many of the chest and those causing fracture might be evacuated after forty-eight hours without injury.

4. The advantages of the pre-operative train are : a ready evacuation and relief of congestion at the front and the opportunity of temporizing in doubtful cases. The disadvantages of the train are : the lack of equipment for caring for the wounded and for feeding them en route, and especially the care of those wounded improperly selected.

VII. Chest Surgery.

1. The indications for operation at the front in chest wounds are : open sucking wounds, hemorrhage, the removing of splintered bone and large foreign bodies. All chest wounds would best be primarily closed.

2. The indications for operation at the base are : empyema, removal of foreign bodies, and aspiration, after two weeks time, of blood in the chest.

3. The most practicable anesthesia after all is likely ether anesthesia or ether carefully induced by chloroform. The use of nitrous oxide, oxygen with the idea of inflating the lungs, we consider a disadvantage, since hemorrhage is frequently controlled on the simple collapse of the lung on entering the pleural cavity. Important in the technic of war surgery of the chest is the position enabling the patient to use the uninjured lung as much as possible. It is rare that the chest can be most advantageously opened through the classical route by resection of the fourth rib in front. It is the proper technic to follow the tract of the missile and thus be able to clean away all debris and devitalized tissue down to the foreign body.

4. After operations on the chest the patient should be propped up, the chest aspirated after ten days if fluid remains, and the wound left closed unless the fluid becomes purulent and the patient septic, when a thoracotomy should be done for drainage.

VIII. Secondary Hemorrhage.

1. Secondary hemorrhage occurs almost always in septic cases.

2. The bleeding vessel should be ligated at a secure point above the sight of bleeding. Time is lost by otherwise temporizing with local ligation and packings.

3. Predisposing causes of secondary hemorrhage are failure to tie proper knots, lack of fixation of compound fractures of limbs, improper selection of ligature material when Dakin solution is to be used, pressure and erosion of drain tubes, and, above all, sepsis.

4. The general treatment of secondary hemorrhage is replacement of blood loss by blood transfused, plenty of water to drink, and ample feeding.

IX. Knee Joints.

1. Non-operative cases of through-and-through machine gun and rifle wounds of the knee joint compare most favorably with operative results, and these cases should not be immediately operated upon.

2. In debridement of the knee would advise in scale of preference complete closure, closure of capsule and fascia, and leaving the wound wide open.

3. Due to the structure of the head of the tibia, its honeycombed cancellous bone tissue, the shattering of the head of the tibia is more serious than the shattering of the condyles of the femur. Sepsis here and infections of this bone tissue have been very severe and stubborn.

4. Knee injuries with marked bone shattering, those containing foreign bodies, and those septic demand immediate amputation.

5. A long continued streptococcus infection of the knee usually ends in amputation.

6. Our attitude has been one of extreme conservatism regarding amputation and our results in cases finally amputated would suggest that we had erred on the side of conservatism when finally we did amputate. In our effort not to amputate at all we have had the tendency to wait too long at the sacrifice of a man's general condition.

7. There are numerous cases of excision of the patella showing good function, but the tendency is to a weakened knee and genu recurvatum. With the patella lost as fulcrum of the long lever of the extensor muscles and the tendon above the patella and the short arm of the tendon below, the patella ligament serves with difficulty the function of extension unless the knee is thrown in a back curve and the ligament allowed to act as a string to a bow. Compared to the normal the function is far from good.

8. Knee joints should be mobilized as soon as possible. The difficulty, however, of forcing the patients to use a painful joint is very great. We have had little success in mobilizing the particular joints at this base due to their septic condition and their severity.

9. All strong antiseptics should be barred from use in the treatment of infections and wounds of the knee joint.

X. Antiseptics.

1. Chemical antisepsis, especially Dakin solution, has shown itself of great value as a cleansing agent in ridding the wound of necrotic tissue, slough, and secretions, and undoubtedly of value in reducing the dose of bacteria to a minimum. Those wounds treated with Dakin solution were sooner closed and with better results than those simply dressed without antiseptics.

2. Antiseptics listed in order of their availability at the front are : iodine, mercury salts, alcohol, and Dakin solution; and at the base : all of these antiseptics with a ready supply of Dakin solution.

XI. Anesthetics.

1. Our nurses have served most efficiently as anesthetists in administering gas oxygen, ether and chloroform. The only objections to an efficient nurse anesthetist are her natural physical weaknesses as compared with a man, and her inability to assist in certain emergencies where the man and physician might excel, coupled with certain inconveniences brought about through a consideration of her sex.

2. Local anesthesia has proved successful and of considerable advantage in head cases, in operations where inhalation anesthesias are contra-indicated on account of infections or affections of the respiratory tract, in closures of small wounds, and in dental surgery. We have always performed thoracotomy under local anesthesia in our cases of empyema. Spinal anesthesia is advantageous in cases of shock, especially combined with gas oxygen or a preparation with morphia and hyoscin. In this way not only may a satisfactory anesthesia, especially below the waist, be obtained, but at the same time the conditions of an anoci-association may be cared for.

3. Gas oxygen and gas oxygen combined with light ether is the anesthetic of choice in shock cases and depleted cases. The period of recovery is certainly lightened in such cases. We consider it contra-indicated in chest cases with a chance of hemorrhage.

4. Gas oxygen has offered a ready and easy induction and relieved excitement where cases were gathered together in the rush of the operating room. The ready induction also allows an immediate beginning of preparation for the operation without the consciousness of the patient. Chloroform though certainly far more dangerous can be used most practicably as an induction to ether.

5. We have had no experience with Depage anesthesia or other cholonic anesthesias and we feel opposed to this form of anesthesia on the principle of indefinite doses and inability to recover an overdosage.

XII. Fluids.

1. Sodium bicarbonate in two per cent. solution certainly raises the blood pressure more quickly and more actively than salt solution. Its effect is likely less transient.

2. Saline given intravenously has been of value in restoring the volume of blood lost through hemorrhage and in temporarily raising the blood pressure, but this effect is soon lost. When a large volume of blood has been lost the result is very temporary and very apt not to hold the patient through the emergency. Water by rectum is absorbed as readily if not more readily than saline subcutaneously. For the continued effect saline intravenously should be followed with water by rectum and by mouth.

3. Gum salt should take an intermediary position between saline and blood.

4. Our experience with gum salt at the front leaves nothing to recommend it. Saline intravenously, in our hands, has offered better results than even gum salts. We believe that gum salt does not keep well and that there is actually some poison, likely of a protein nature, produced to account for cases of death after its administration. There is nothing to equal blood itself in shock and hemorrhage.

XIII. Blood Transfusion.

1. Citrated blood given through the intermediary of a large ampule under light pressure; the same ampule used for receiving and giving the blood. Our method is to prepare a large sterile ampule of 500 cc. capacity drawn to a tube at both ends and containing sealed up 3 1/2 grams of sodium citrate solution. One end is connected with a short rubber tube and needle, and the other end with a rubber tube into which a suction or pressure pump can be inserted. The blood is taken from the donor under negative pressure, shaken as it runs with the citrate solution, and then delivered to the recipient under light positive pressure. This method offers an already prepared sterile apparatus and an easy means of manipulation in the hands of any operator. The total apparatus and solution is kept sterile and ready for immediate use.

2. We have had neither hemolytic nor protein reactions in our cases of blood transfusion. They have all been carefully grouped.

3. We have had good success in cases of prolonged infections both immediate and remote.

4. Available sources of blood are gassed cases and lightly wounded. We do not recommend using our personnel. The difficulty of finding donors has never been experienced at the Base as at the front. The only source of blood recommended at the Base is from properly grouped healthy donors, and we have had no trouble in finding sufficient volunteers.

XIV. Amputations.

1. The value of the guillotine operation is that it is quick, conserves the part and offers an open surface free from pockets for drainage and the access of antiseptics.

2. The medio-tarsal amputation is not justifiable in war surgery. There should be no compromise in disarticulations, resections and attempt at bone plastics in infected war vounds.

3. The lower third amputation is preferable to an attempt to conserve by Symes operation.

4. Would not compromise amputation by a consideration of the terminal scar in war surgery. Where conditions are favorable would seek a lateral flap for stumps of the lower extremities and terminal scar for the upper extremities.

5. Do not recommend amputations through the knee joint.

6. Bones of stumps finally cover with fibrous and bony callous; they round off and actually become drum sticks. It is just as well that they be left parallel and time not lost in attempting to shape them.

7. A stump of four or five inches should be left below the knee.

8. A stump of at least three inches should be left below the elbow.

9. Amputations should not be made through the elbow joint.

XV. Head Injuries.

1. All laceration of the scalp should be explored for fracture since fluoroscopic reports on the skull are very apt to be deceptive.

2. Foreign bodies in the brain should be accurately localized and removed. No drainage at the primary operation should be left down into the brain.

3. The magnet is useful in extracting foreign bodies from the brain, and allows extraction without the damage done by the opening and closing of bullet forceps or other instruments. The manipulation of such another instrument through the uncertainty of its grasp is apt to do considerable damage to the brain substance.

4. We have seen several late abscesses of the brain which, in every case except one, followed the leaving of the foreign body and debris in place. They did well after secondary opening without drainage.

XVI. Hospital Problems.

1. Base hospitals should be located as near as possible to the evacuation hospitals, and an ample and quick line of communication established between them. In this way the evacuation of patients could be expedited and many cases transferred to the base where first attention could be given and where they could remain until sent to Rest Camps. There are many cases which would actually benefit by this time of observation before any operation is determined upon, and just as many who would benefit by resting at the same place where first operated.

The arrangement of the hospital should be as compact as possible in a ward system rather than the widespread and loosely strung barrack system connected by long corridors or open bridges.

The accompanying plan of our surgical service will show an arrangement of the service fitted into the administration department. This plan has met every emergency. Also the enclosed "Memorandum to Ward Surgeons" will indicate roughly the operation of this plant.

The number and quality of the personnel at the Base should not be sacrificed for the sake of the front alone. During the development of our service almost all of the officers on the surgical staff were young men and the staff was augmented temporarily from time to time by a few transient casual officers. At one time 800 surgical cases were cared for by six officers, and at another time 2,200 cases occupying 55 wards were looked after by twelve officers on the staff, assisted by six casual officers and two officers lent by the medical service. Our personnel present has always been in inverse proportion to the amount of work on hand.

The Commanding Officer should be a regular army officer or an efficient hospital administrator whose duties should be given over entirely to administration, and his assistants in the administration department should not be chosen from among physicians and surgeons whose aptitude for such work is proverbially bad and whose services where they are really fit would be most effective. Questions of purely a medical or surgical nature should be left entirely to the heads of the medical and surgical services, and they should be allowed freedom in carrying out their judgment of medical and surgical problems. They should act as advisors to the Commanding Officer on such medical and surgical questions, and he, as advisor to them, solely on questions of administration.

2. The Surgical Team is elastic and should be efficient in its scope of work if properly chosen as to its efficiency and co-operation.

3. A Mobile Hospital should be small enough to be readily moved, even to the position of the Field Hospital. My impression is that the existing Mobile Hospitals were too large and unwieldy.

4. Its equipment should be ample to take care of all emergencies and nothing more.

5. The function of the Mobile Hospital should be to take care of non-transportables and as far forward as possible in the zone of advance. Their great function is to go where the concentration of work demands them.

6. It is best to have the special branches of surgery gathered in wards of a general hospital for the sake of the co-operation and consultation of the various departments and their use of the laboratories, etc. There is a great danger in extreme specialization without consultation.

7. Our hospital was not sufficiently supplied with instruments either of quality, types, or quantity. All efforts to obtain them from the Army were vain and our demand in emergency was only supplied by instruments brought with the Base Hospital units and purchased from French instrument makers from a fund carried by the Chief of the Surgical Service.

There was no lack of dressings, bandages, and splints of all kinds, supplied by the Base Hospital units and the American Red Cross.

---

BASE HOSPITAL N° 6.

